# Mechanisms Involved in Nicotinic Acetylcholine Receptor-Induced Neurotransmitter Release from Sympathetic Nerve Terminals in the Mouse Vas Deferens

**DOI:** 10.1371/journal.pone.0029209

**Published:** 2011-12-22

**Authors:** Damian J. Williams, Peter Sidaway, Thomas C. Cunnane, Keith L. Brain

**Affiliations:** 1 Department of Pharmacology, University of Oxford, Mansfield Road, Oxford, United Kingdom; 2 Neuropharmacology and Neurobiology, School of Clinical and Experimental Medicine, University of Birmingham, Birmingham, United Kingdom; University of Houston, United States of America

## Abstract

Prejunctional nicotinic acetylcholine receptors (nAChRs) amplify postganglionic sympathetic neurotransmission, and there are indications that intraterminal Ca^2+^ stores might be involved. However, the mechanisms by which nAChR activation stimulates neurotransmitter release at such junctions is unknown. Rapid local delivery (picospritzing) of the nAChR agonist epibatidine was combined with intracellular sharp microelectrode recording to monitor spontaneous and field-stimulation-evoked neurotransmitter release from sympathetic nerve terminals in the mouse isolated vas deferens. Locally applied epibatidine (1 µM) produced ‘epibatidine-induced depolarisations’ (EIDs) that were similar in shape to spontaneous excitatory junction potentials (SEJPs) and were abolished by nonselective nAChR antagonists and the purinergic desensitizing agonist α,β-methylene ATP. The amplitude distribution of EIDs was only slightly shifted towards lower amplitudes by the selective α7 nAChR antagonists α-bungarotoxin and methyllcaconitine, the voltage-gated Na^+^ channel blocker tetrodotoxin or by blocking voltage-gated Ca^2+^ channels with Cd^2+^. Lowering the extracellular Ca^2+^ concentration reduced the frequency of EIDs by 69%, but more surprisingly, the Ca^2+^-induced Ca^2+^ release blocker ryanodine greatly decreased the amplitude (by 41%) and the frequency of EIDs by 36%. Ryanodine had no effect on electrically-evoked neurotransmitter release, paired-pulse facilitation, SEJP frequency, SEJP amplitude or SEJP amplitude distribution. These results show that activation of non-α7 nAChRs on sympathetic postganglionic nerve terminals induces high-amplitude junctional potentials that are argued to represent multipacketed neurotransmitter release synchronized by intraterminal Ca^2+^-induced Ca^2+^ release, triggered by Ca^2+^ influx directly through the nAChR. This nAChR-induced neurotransmitter release can be targeted pharmacologically without affecting spontaneous or electrically-evoked neurotransmitter release.

## Introduction

Activation of nAChRs located on nerve terminals is an important mechanism that modulates neurotransmitter release. Prejunctional nAChRs are involved in fundamental aspects of synaptic plasticity [1] and alterations of nAChR function have been implicated in a number of disease states including Parkinson's Disease, Alzheimer's Disease and drug dependence [2], [3]. Activation of nAChRs located on postganglionic sympathetic nerve terminals is associated with the pathological effects of smoking on the heart [4]. Most research on the mechanism of nAChR-induced modulation of neurotransmitter release has been carried out in the CNS, where a number of mechanisms have been proposed including initiation of action potentials [5], activation of voltage-gated Ca^2+^ channels [VGCCs; 6], direct influx of Ca^2+^ through the nAChR [7], mobilisation of intraneuronal Ca^2+^ stores [8], an unidentified NO-dependent process [9] and a number of Ca^2+^-dependent cellular processes, such as recruitment of protein kinases [see 10].

The rodent vas deferens is richly innervated by sympathetic nerves [11] and provides a model system to study sympathetic neurotransmission. Previous studies in the rodent vas deferens have shown that activation of nAChRs can both induce neurotransmitter release and potentiate electrically-evoked neurotransmitter release [12], [13], [14], [15], although the precise mechanism of the modulation remains unknown.

The aim of the present inquiry was to investigate the mechanism of nAChR-induced neurotransmitter release in mouse vas deferens using the potent nAChR agonist epibatidine [16]. A method of rapid local application of epibatidine was used to avoid variability in response, likely caused by desensitisation of nAChRs, that occurs when using a slower, bath-applied approach [15]. We show that the majority of epibatidine-induce neurotransmitter release occurs following influx of Ca^2+^ directly through non-α7 nAChRs. This Ca^2+^ influx triggers Ca^2+^-induced Ca^2+^ release (CICR) from intraterminal stores, which leads to further neurotransmitter release.

## Materials and Methods

### Ethics statement

All experiments were carried out in accordance with the guidelines of the UK Animal (Scientific Procedures) Act 1986. As these experiments did not involve regulated procedures (as defined by the Act described above), institutional review board or ethics committee approval was not required. However, independent academic oversight was provided by the academic in charge of animal use, Dr S. Totterdell (Department of Pharmacology, Oxford).

### Tissue preparation

Vasa deferentia were removed from 8–12 week-old Balb/c mice which were killed by cervical dislocation. The prostatic quarter of each vas deferens was removed to ensure that no sympathetic ganglia were present in the preparation. The bathing physiological saline solution (PSS) contained (mM): 118.4 NaCl, 25.0 NaHCO_3_, 1.13 NaH_2_PO_4_, 1.8 CaCl_2_, 4.7 KCl, 1.3 MgCl_2_ and 11.1 glucose. The solution was gassed with a mixture of 95% O_2_ and 5% CO_2_ to pH 7.4 and maintained at a temperature of 35–37°C.

### Electrophysiological studies

Conventional intracellular recording techniques were used to monitor membrane potentials in individual smooth muscle cells. The vas deferens was carefully pinned to the Sylgard (Dow-Corning, UK) covered base of a 5 ml Perspex organ chamber perfused with PSS at a rate of 2 ml per minute. The membrane potential of individual smooth muscle cells close to the surface of the vas deferens was measured using a sharp microelectrode. Microelectrodes were connected by an Ag/AgCl wire to the input headstage of an Axoclamp 2B (Axon Instruments, USA). The data were digitized (1 kHz sampling) using a PowerLab 4SP (AD Instruments, UK) and recorded on a G4 computer (Apple) with Chart 5 software (AD Instruments, UK). Microelectrodes were fabricated from borosilicate glass tubing containing an inner glass filament (outer diameter 1.5 mm, inner diameter 0.86 mm; Clark Electromedical, USA) using a Flaming-Brown P87 electrode puller (Sutter Instruments, USA). The microelectrodes were filled with 5 M potassium acetate and had tip resistances of 30 to 90 MΩ.

Spontaneous excitatory junction potentials (SEJPs) and epibatidine-induced depolarisations (EIDs) were automatically detected using the ‘template’ function of Chart 5. This function uses a correlation algorithm to compare the trace with a selected typical SEJP in a given experiment. The template was redefined for each cell. The algorithm applies a rolling normalisation so that the correlation coefficient was independent of the baseline and amplitude of any SEJP/EID. Trace segments with correlation coefficients of >0.8 (where 1 is an exact match) and amplitudes of >2 mV were counted as SEJPs/EIDs; below an amplitude of 2 mV, SEJPs/EIDs were difficult to distinguish from baseline noise.

Electrical stimuli (pulse width 0.1 ms, 15 V amplitude) were delivered through a pair of platinum electrodes positioned around the prostatic end of the vas deferens. The stimuli were generated by a digital stimulator (Applegarth Instruments, Oxford) coupled to an optically-isolated stimulus unit. Excitatory junction potential (EJP) amplitude was measured from the resting membrane potential (RMP) to the peak amplitude following field stimulation. The RMP was calculated by averaging the membrane potential 100 ms prior to stimulation.

### Rapid application of epibatidine

Working epibatidine solution (1 µM) was prepared in PSS and passed through a 0.22 µM nitrocellulose filter (Millipore) to remove any particulate matter before loading into a micropipette. The micropipette was positioned close to the surface of the vas deferens immediately adjacent to the intracellular microelectrode. The micropipettes were fabricated using a Flaming-Brown P87 electrode puller. Epibatidine was ejected from the tip of the micropipette by a brief (50–200 ms) pulse of compressed nitrogen gas (4 MPa) controlled by a Picospritzer (General Valve Corp., USA). The volume of the drug ejected from the micropipette was ≤100 pl. Ejection of PSS alone had no detectable effects.

### Data analysis

Data were analysed using Prism 4 software (GraphPad, USA) and Mini Analysis Program (Synaptosoft, USA). When measuring SEJP repolarization time course, the time taken for the membrane potential to recover by a factor of 1/e from 90% of the peak amplitude was measured using the ‘Peak Parameters’ function of Chart (ADInstruments, Oxford, UK) and excluding events where two peaks occurred with the measurement window (of 100 ms). In order to avoid template matching biasing the shape of the repolarisation, EID/SEJPs were automatically detected by detecting a local peak in the dV/dt above 200 mV.s^−1^ (i.e. only on the basis of the rising phase). As the time course varied amongst preparations, a 2-way ANOVA was used to separate the effect of inter-preparation variation from epibatidine-induced variation. For analysis of EID/SEJP amplitude distributions, Kolmogorov-Smirnov tests were used. Elsewhere, a paired or unpaired (as appropriate) Student's *t*-tests were applied to determine statistical significance. A *P*<0.05 was considered significant.

### Drugs

Stock solutions of d-tubocurarine (chloride hydrate), hexamethonium (chloride), α- bungarotoxin, α,β-methylene ATP (lithium salt), tetrodotoxin (citrate) and CdCl_2_ were dissolved in distilled water. Epibatidine (hydrochloride) and ryanodine were dissolved in DMSO. The maximum final concentration of DMSO was 0.1%. All solutions were prepared and aliquotted before storing at −20°C. All drugs passed through a maximum of one freeze-thaw cycle. Epibatidine, ryanodine, tetrodotoxin and α-bungarotoxin were obtained from Tocris, UK; all other drugs were obtained from Sigma-Aldrich, UK. The drug vehicle alone was without effect.

## Results

### The effect of locally-applied epibatidine on SEJPs and EJPs

The resting potential of smooth muscle cells in the mouse vas deferens was monitored by intracellular recording before and during the focal application of 1 µM epibatidine from a micropipette positioned above the surface of the tissue, adjacent to the recording microelectrode. Multiple epibatidine responses could be recorded from a single preparation by repositioning the electrode and micropipette to different areas of the vas deferens for each epibatidine application.

**Figure 1:**
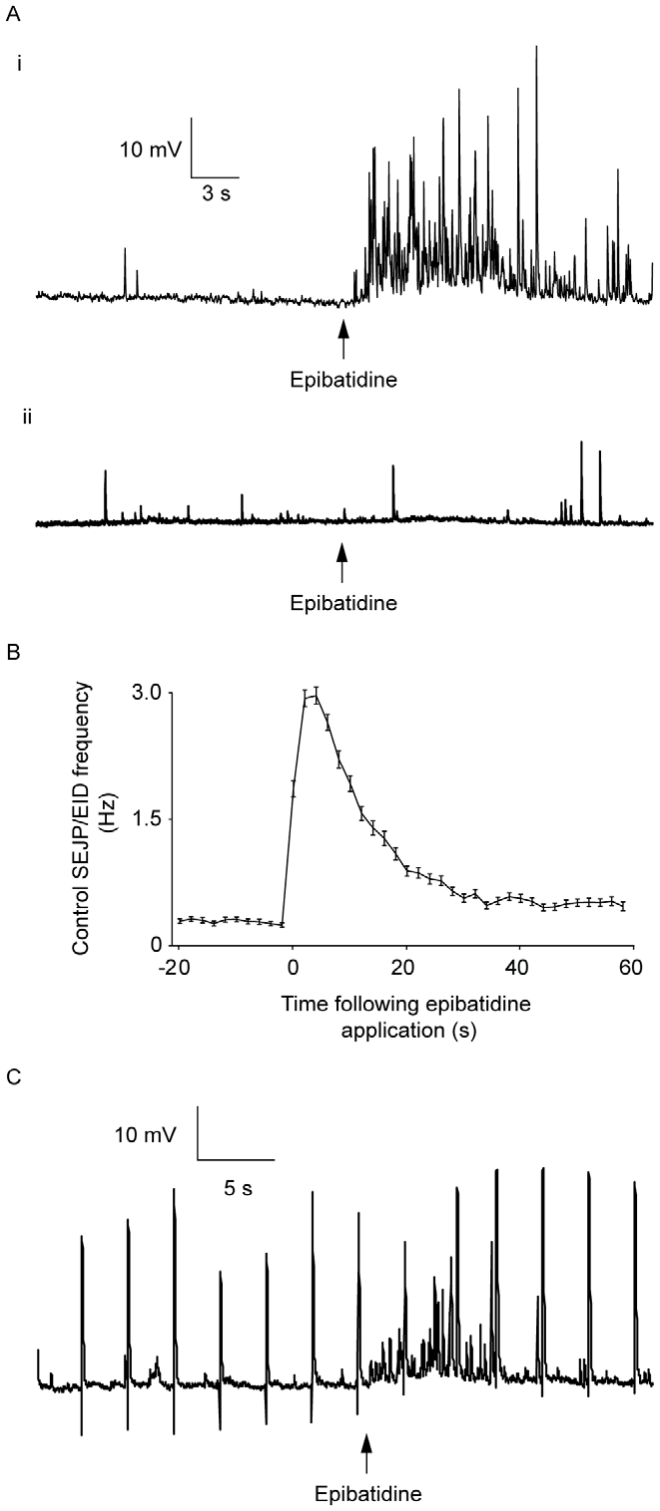
The effect of rapid application of epibatidine (1 µM) on the membrane potential of smooth muscle cells in mouse isolated vas deferens. (a) Typical traces showing membrane potentials before and after the local application of 1 µM epibatidine. RMPs were between −70 and −80 mV. (i) Most (71±3%) cells displayed clear depolarisations following epibatidine application. These depolarisations were termed ‘epibatidine-induced depolarisations’ (EIDs) (ii) In 25±2% of cells, application of epibatidine had no observable effect. The time of epibatidine application is marked with an arrow (b) Graph showing the effect of local application of epibatidine on the frequency of SEJP/EID occurrence. The points show mean ± s.e.m. of the frequency of junction potentials collected in 2 s bins. Only data from cells that responded to epibatidine were plotted (*n_c_* = 227). (c) The effect of local application of epibatidine on electrically-evoked neurotransmitter release. A representative membrane potential trace showing EJPs before and after epibatidine application. EJPs were evoked by electrical stimulation at a frequency of 0.33 Hz.

Following local application of 1 µM epibatidine, a large number of rapid depolarisations of the membrane occurred, similar in appearance to SEJPs (Fig. 1Ai; for a different example, shown at higher resolution, see Fig. S1). Just like SEJPs, these depolarisations had a rapid rising phase, followed by a slower repolarization (fitted time constant of repolarisation of control SEJPs: 25.8 [22.1–31.6] ms; following epibatidine: 26.5 [20.2–31.4] ms; median [inter-quartile range]; *n* = 418 events, number of cells (*n_c_*) = 12, number of vasa deferentia (*n_v_*) = 3; 2-way ANOVA *P* = 0.47). Despite such similarities, these events will be referred to as epibatidine-induced depolarisations (EIDs) to distinguish them from true spontaneous neurotransmitter release (SEJPs). EIDs occurred in 72±3% of smooth muscle cells following the local application of epibatidine. In 24±2% of cells, epibatidine had no obvious effect (Fig. 1Aii). In the remainder of recordings, the microelectrode was displaced from the cell and no useful data were obtained (*n_c_* total = 330; *n_v_* total = 54). In the cells that responded to epibatidine, the frequency of EID occurrence reached a maximum at around 6 s after epibatidine application and then returned back towards the control level (Fig. 1B). The response to epibatidine was somewhat variable (see Fig. S2 for example traces): the mean frequency of EIDs in the 20 s following epibatidine was 1.99 Hz with a standard deviation of 0.95 Hz (*n_c_* = 227). Experiments were attempted using a lower concentration of epibatidine (100 nM) but no consistent effects were observed (*n_v_* = 3; results not shown).

The effect of rapid, locally-applied epibatidine on electrically-evoked neurotransmitter release was also investigated. EJPs were evoked at a frequency of 0.33 Hz by single field stimuli. The mean EJP amplitude in the 20 s immediately following epibatidine application was 118±6% of the mean control EJP amplitude measured in the 20 s prior to epibatidine addition (Fig. 1C; *P*<0.05, paired Student's *t*-test; *n_c_* = 18, *n_v_* = 4). The variability of EJP amplitude was not significantly different after epibatidine addition (control mean EJP variance = 32±8 mV^2^, epibatidine mean EJP variance = 40±10 mV^2^; P = 0.32, paired Student's *t*-test). These results suggested that rapidly applied epibatidine can cause a small but significant increase in electrically-evoked neurotransmitter release.

**Figure 2:**
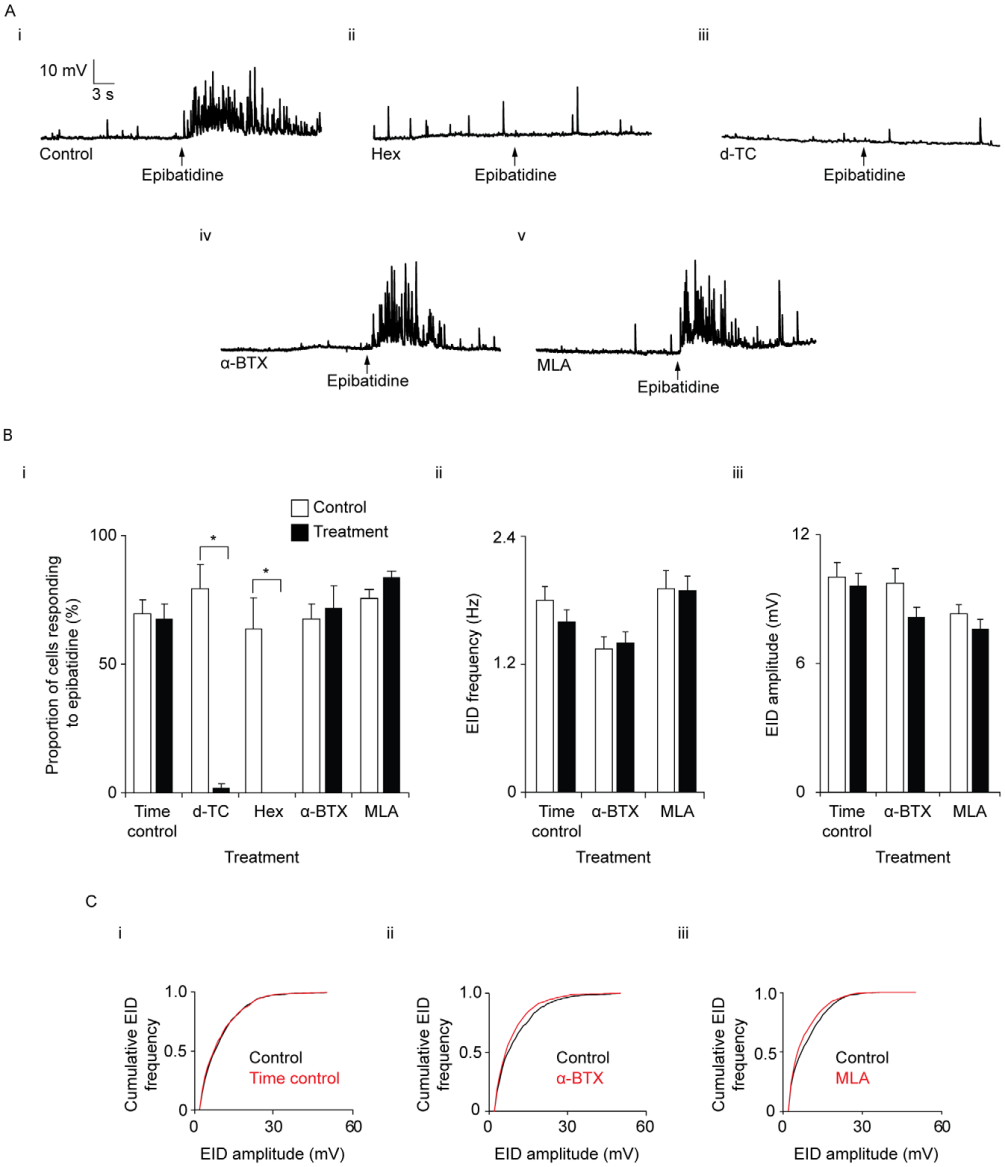
The effect of nAChR antagonists on EIDs. A) Representative traces of smooth muscle cells showing local application of epibatidine in presence of nAChR antagonists for 60 minutes. (i) 60 minute temporal control (ii) hexamethonium (Hex; 100 µM) (iii) or d-tubocurarine (100 µM; d-TC) (iv) α-bungarotoxin (α-BTX; 100 nM) (vi) methyllycaconitine (20 nM; MLA). RMPs were between −70 and −80 mV. B) Quantification of the effects of nAChR antagonists on EIDs. In each chart, two bars are shown for each drug: control epibatidine applications (outline) and epibatidine applications following treatment (filled). Each bar represents the mean ± s.e.m. (i) bar chart showing the mean percentage of cells that responded to the local application of epibatidine. (ii) A bar chart showing the mean frequency of EID occurrence in cells which responded to epibatidine. (iii) A bar chart showing the mean amplitude of EIDs in responding cells. C) Cumulative frequency plots of EID amplitudes in (i) time control experiments and in the presence of (ii) α-BTX (iii) MLA. There was no significant change in the EID amplitude distribution in the time control experiments. In the α-BTX experiments, there was a significant shift to smaller amplitudes.

To determine the mechanism of EIDs, a number of single epibatidine applications and recordings were made from different areas of the same preparation in the presence and absence of a pharmacological agent. A series of time controls were carried out to ensure that any changes to the properties of EIDs were not caused by nAChR desensitization or a run down in response that may have occurred during the period of drug incubation. For these experiments, a series of control applications of epibatidine were carried out and, after 60 minutes in the absence of epibatidine-application, a second series of epibatidine application and recordings were made (Fig. 2Ai). There was no significant difference between the mean fraction of cells that responded to the initial application of epibatidine (70±5%) and the fraction of cells that responded to epibatidine after 60 minutes (67±6%; *P* = 0.84; *n_c_* control = 44, *n_c_* time control = 46, *n_v_* = 6; Fig. 2Bi). In the responding cells, there was no significant change in EID frequency of occurrence between control epibatidine applications and time controls (control frequency = 1.20±0.09 Hz, time control frequency = 1.16±0.09 Hz; *P* = 0.77, *n_c_* control = 29, *n_c_* time control = 30, *n_v_* = 6; Fig. 2Bii), nor mean amplitude (10.1±0.6 mV compared with 9.7±0.5 mV; *P* = 0.65; Fig. 2Biii), nor amplitude distribution (*P* = 0.10, Fig. 2Ci).

### The effect of nAChR antagonists on EIDs

To confirm that EIDs occurred as a result of activation of nAChRs, experiments were carried out in the presence of nAChR antagonists. Following exposure to the nonspecific, noncompetitive neuronal nAChR antagonist hexamethonium (100 µM) for 60 minutes, EIDs were abolished: 63±12% of the cells responded to 1 µM epibatidine in the control, and following exposure to hexamethonium, no cells responded to epibatidine (*n_c_* control = 35, *n_c_* hexamethonium = 40, *n_v_* = 4; Fig. 2Aii, 2Bi). In the presence of the non-specific competitive nAChR antagonist d-tubocurarine (100 µM) for 60 minutes, only one cell responded to epibatidine (*n_c_* control = 40, *n_c_* d-tubocurarine = 51, *n_v_* = 5; Fig. 2Aiii, 2Bi, responding cell not shown).

In order to determine the role of nAChR containing α7-subunits in EID generation, experiments were carried out in the presence of 100 nM α-bungarotoxin (α-BTX) or 20 nM methyllcaconitine (MLA). In the presence of α-BTX (100 nM) for 60 minutes (Fig. 2iv), there was no significant change in the proportion of cells that responded to epibatidine (67±6% in the controls, 72±8% in the presence of α-BTX; *P* = 0.73; *n_c_* control = 46, *n_c_* α-BTX = 45, *n_v_* = 5; Fig. 2Bi). α-BTX had no significant effect on the frequency of occurrence of EIDs (0.90±0.08 Hz in the control, 0.93±0.07 Hz in the presence of α-BTX; *P* = 0.73; *n_c_* control = 30, *n_c_* α-BTX = 31, *n_v_* = 5, Fig. 2Bii). The average amplitude of EIDs in the control was 9.7±0.7 mV and 8.2±0.5 mV in the presence of α-BTX (*P* = 0.05; Fig. 2Biii). There was a significant shift in EID amplitude distribution to smaller amplitudes in the presence of α-BTX (*P*<0.05; Fig. 2Cii), but there was no obvious change in the time course of EID occurrence (Fig. S3). Following exposure of the vasa deferentia to 20 nM MLA for 60 minutes (Fig. 2Av), there was no significant change in the proportion of cells (76±3% in the controls, 84±2% in the presence of MLA; *P* = 0.15; *n_c_* control = 30, *n_c_* MLA = 30, *n_v_* = 5; Fig. 2Bi). MLA had no significant effect on the frequency of occurrence of EIDs (1.91±0.17 Hz in the control, 1.90±0.13 Hz in the presence of MLA; *P* = 0.93; *n_c_* control = 30, *n_c_* MLA = 31, *n_v_* = 5, Fig. 2Bii). The average amplitude of EIDs in the control was 8.3±0.4 mV and 7.6±0.4 mV in the presence of MLA (*P* = 0.25; Fig. 2Biii). There was a significant shift in EID amplitude distribution to smaller amplitudes in the presence of MLA (*P*<0.05; Fig. 2Ciii). These findings suggest that the majority of EIDs occur as a result of activation of nAChRs that do not contain α7 subunits.

### The effects of α,β-methylene ATP, tetrodotoxin, Cd^2+^ and reduced extracellular Ca^2+^ concentration on EIDs

**Figure 3:**
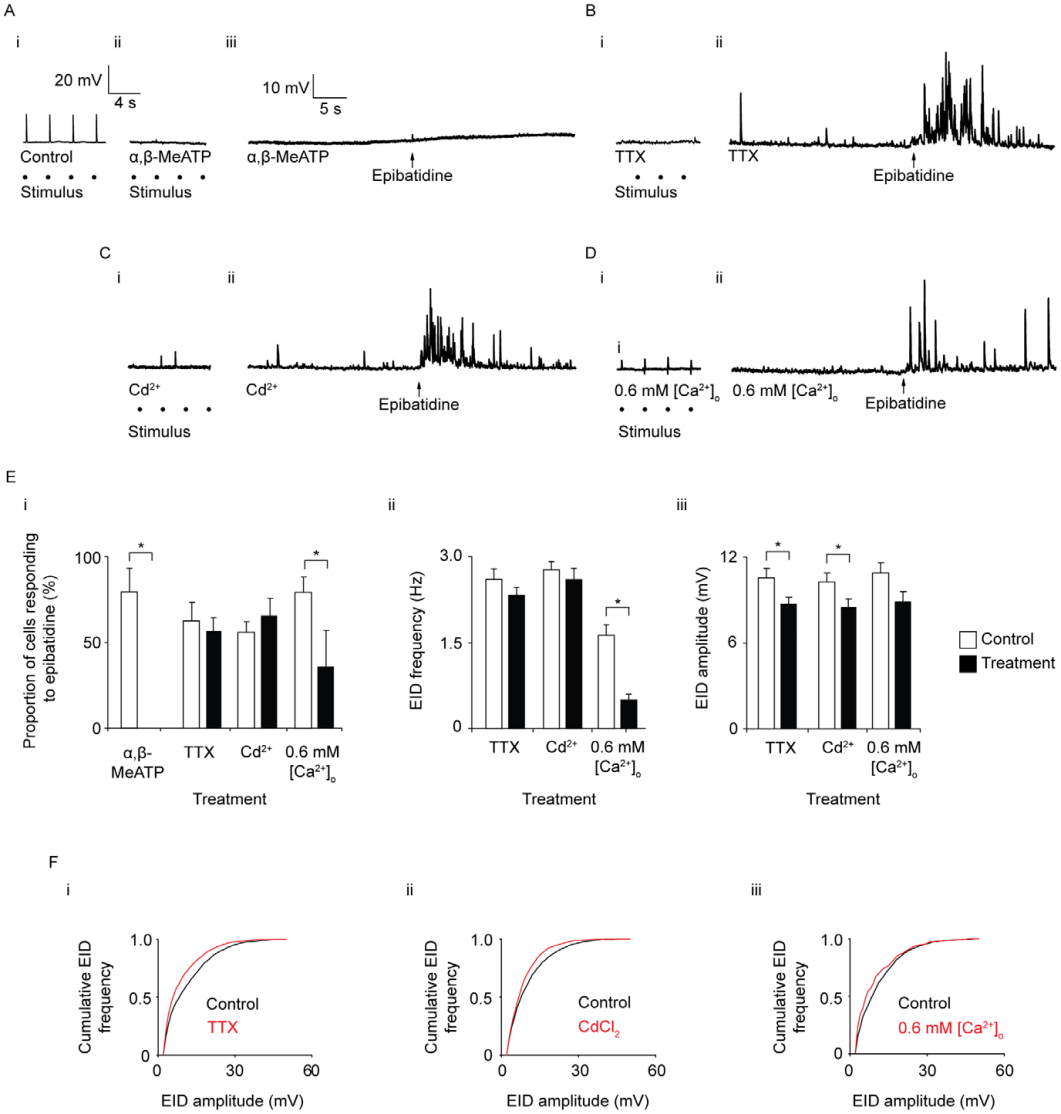
The effect of α,β-Methylene ATP, tetrodotoxin, Cd^2+^, or reduced extracellular Ca^2+^ concentration on EIDs. Representative membrane potential traces before and after epibatidine application, in the presence of (A) α,β-MethyleneATP (α,β-MeATP; 1 µM), (B) tetrodotoxin (TTX; 300 nM), (C) Cd^2+^ (100 µM), (D) 0.6 mM extracellular Ca^2+^ concentration (0.6 mM [Ca^2+^]_o_), each for 60 minutes. The effect of each treatment on electrically-evoked neurotransmitter release is shown in the smaller trace in each section. EJPs were evoked by neuronal stimulation at 0.33 Hz. (Ai) shows EJPs in a representative control trace, (Aii) in the presence of α,β-MeATP, (Bii) TTX, (Cii) CdCl_2_ (Dii) 0.6 mM [Ca^2+^]_o_. RMPs were between −70 and −80 mV. (E) Quantification of EIDs in the presence of α,β-MeATP, TTX, CdCl_2_, or 0.6 mM [Ca^2+^]_o_. In each chart, two bars are shown for each treatment: control epibatidine applications (outline) and epibatidine applications following treatment (filled). Each bar represents the mean ± s.e.m. (i) Bar chart showing the mean percentage of cells that responded to epibatidine. (ii) Bar chart of mean EID frequency. (iii) Bar chart of mean EID amplitude.” (F). Cumulative frequency plots of EID amplitude in the presence of (i) TTX, (ii)Cd^2+^ (iii) 0.6 mM [Ca^2+^]_o_. All treatments caused a significant shift of EIDs to smaller amplitudes (*P*<0.05, Kolmogorov-Smirnov test).

From the above results, it is likely that the EIDs result from activation of prejunctional nAChRs causing neuronal release of ATP. To confirm that the depolarizations were due to activation of postjunctional P2X1 receptors, preparations were incubated with α,β-methylene ATP (α,β-MeATP) for 60 minutes. In vasa deferentia exposed to 1 µM α,β-MeATP, EJPs were abolished (Fig. 3Ai and 3Aii). EIDs were also abolished by α,β-MeATP: in control recordings in the absence of α,β-MeATP, 80±13% of the cells responded to epibatidine, whereas in the presence of α,β-MeATP, no cells responded (*n_c_* control = 24, *n_c_* α,β-MeATP = 27, *n_v_* = 3; Fig. 3Aiii and 3Ei).

A potential mechanism of epibatidine-induced neurotransmitter release involves the generation of neuronal action potentials. In this mechanism, depolarisation of the neuron caused by influx of cations through nAChRs leads to the activation of voltage-gated Na^+^ channels. The resulting action potentials cause neurotransmitter release. To investigate this mechanism, experiments were carried out in the presence of the voltage-gated Na^+^ channel blocker tetrodotoxin (TTX), which blocks neuronal action potential initiation and propagation in the rodent vas deferens [17]. Exposure of vasa deferentia to 300 nM TTX for one hour abolished EJPs (Fig. 3Bi) but EIDs could still be elicited (Fig. 3Bii). The proportion of cells that responded to epibatidine in the presence of TTX (63±11%) was not significantly different to the proportion of cells that responded in the control (56±8%; *P* = 0.29; *n_c_* control = 53, *n_c_* TTX = 61, *n_v_* = 6; Fig. 3Ei). The mean frequency of EIDs was not significantly changed between control applications of epibatidine and applications in the presence of TTX (control frequency = 1.74±0.12 Hz, TTX frequency = 1.55±0.09 Hz, *P* = 0.22, *n_c_* control = 29 *n_c_* TTX = 38, *n_v_* = 6; Fig. 3Eii). In the presence of TTX, there was a small but significant decrease in the mean amplitude of EIDs: from 10.5±0.7 mV in the control, to 8.7±0.5 mV in the presence of TTX (*P*<0.05; Fig. 3Eiii). There was also a significant change in EID amplitude distribution in the presence of TTX (*P*<0.05, Fig. 3Fi), with a decrease in the proportion of higher-amplitude EIDs. These results indicated that the majority of EID occurred through a mechanism independent of voltage-gated Na^+^ channel activation.

EIDs may occur as a result of nerve terminal depolarization that not does require voltage–gated Na^+^ channel activation: influx of cations through the nAChR may cause a local depolarization which results in the direct activation of voltage-gated Ca^2+^ channels (VGCCs). To investigate the role of VGCCs in the generation of EIDs, experiments were carried out in the presence of Cd^2+^, a non-specific VGCC blocker. Following exposure to 100 µM Cd^2+^ for 1 hour, EJPs were blocked (Fig. 3Ci), but EIDs could still be induced (Fig. 3Cii). The proportion of cells that responded to epibatidine in the presence of Cd^2+^ was unchanged from control applications (control = 56±7%, Cd^2+^ = 64±10%; *P* = 0.53; *n_c_* control = 57, *n_c_* Cd^2+^ = 50, *n_v_* = 5; Fig. 3Ei). The frequency of EID occurrence was not significantly changed from control in the presence of Cd^2+^: control EID frequency was 1.9±0.1 Hz, and in the presence of Cd^2+^, the frequency of EIDs was 1.7±0.1 Hz (*P* = 0.48; *n_c_* control = 28, *n_c_* Cd^2+^ = 30, *n_v_* = 5 Fig. 3Eii). There was a small but significant decrease in the mean amplitude of EIDs in the presence of Cd^2+^ (control amplitude = 10.2±0.6 mV, CdCl_2_ amplitude = 8.5±0.5 mV; *P*<0.05; Fig. 3Eiii). The EID amplitude distribution was significantly shifted to smaller amplitudes in the presence of Cd^2+^, compared with controls (*P*<0.05, Fig. 3Fii).

It has previously been demonstrated that direct influx of Ca^2+^ through nAChRs into nerve terminals is sufficient to trigger neurotransmitter release [7]. To investigate the role of extracellular Ca^2+^ on the generation of EIDs, experiments were carried out in PSS with reduced Ca^2+^ concentration. An extracellular Ca^2+^ concentration ([Ca^2+^]_o_) of 0.6 mM was chosen because at lower concentrations the resting membrane potential became unstable and it was very difficult to obtain recordings. After incubation of the vasa deferentia in 0.6 mM [Ca^2+^]_o_ PSS for 60 minutes, EJP amplitude was reduced (Fig. 3Di) and the response to epibatidine application was diminished (Fig. 3Dii). The mean proportion of cells that responded to epibatidine in the presence of 0.6 mM [Ca^2+^]_o_ (34±21%) was significantly smaller than the proportion of cells that responded in the control (79±9%; *P*<0.05; *n_c_* control = , *n_c_* 0.6 mM [Ca^2+^]_o_ = 61, *n_v_* = 6; Fig. 3Ei). The mean frequency of EIDs was significantly reduced in the presence of 0.6 mM [Ca^2+^]_o_ (control frequency = 1.08±0.12 Hz, 0.6 mM [Ca^2+^]_o_ frequency = 0.33±0.06 Hz; *P*<0.05; *n_c_* control = 30, *n_c_* 0.6 mM [Ca^2+^]_o_ = 30, *n_v_* = 5; Fig. 3Eii). In the presence of 0.6 mM [Ca^2+^]_o_, there was no statistically significant change in the mean amplitude of epibatidine-induced junction potentials (10.9±0.7 mV in the control; 8.9±0.7 mV in the presence of 0.6 mM [Ca^2+^]_o_; *P* = 0.05; Fig. 3Eii). There was a significant change in EID amplitude distribution in the presence of 0.6 mM [Ca^2+^]_o_ (*P*<0.05, Fig. 3Fiii).

### The effect of ryanodine on EID occurrence, EJPs, and SEJPs

**Figure 4:**
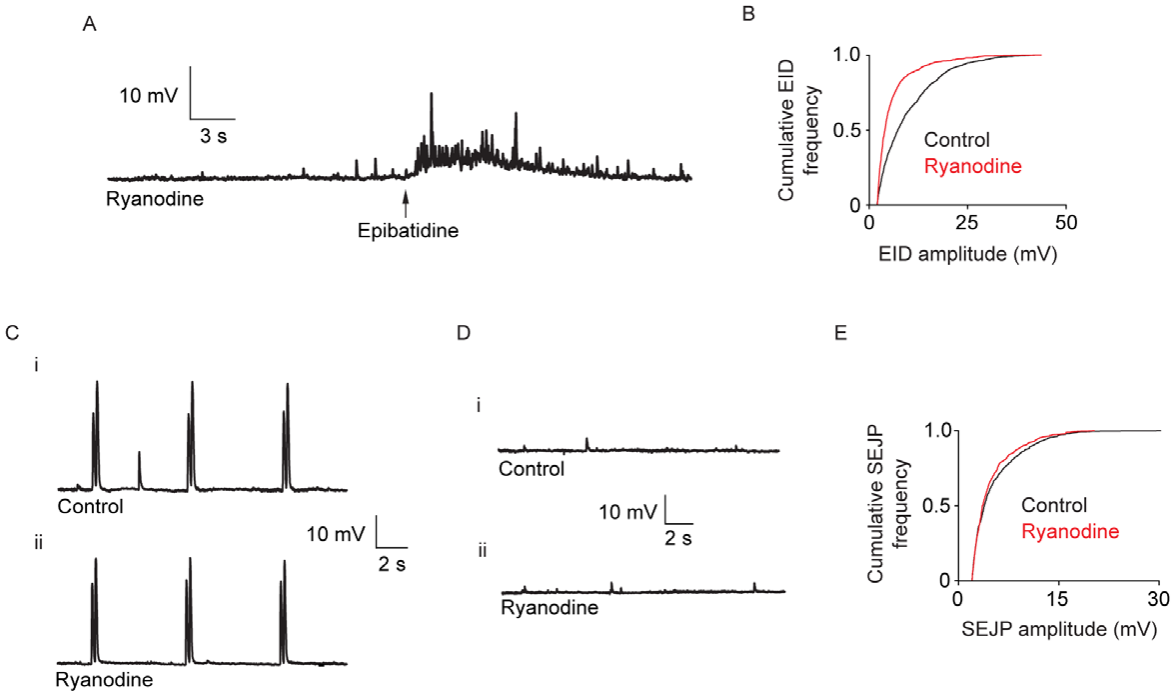
The effect of ryanodine on epibatidine-induced, electrically-evoked, and spontaneous neurotransmitter release. (A) A typical membrane potential trace showing the effects of epibatidine application following exposure to 10 µM ryanodine for 60 minutes. (B) A cumulative frequency plot of EID amplitude in the presence of ryanodine. There is a significant shift to smaller EID amplitudes in the presence of ryanodine (P<0.05, Kolmogorov-Smirnov test. (C) Representative membrane potential trace showing pairs of EJPs evoked by neuronal stimuli 200 ms apart at a frequency of 0.2 Hz (i) in control recordings and (ii) after ryanodine application for 60 minutes. (D) A representative membrane potential trace in the absence of neuronal stimulation showing (i) SEJPs in control and (ii) in the presence of ryanodine. (E) A cumulative frequency plot of SEJP amplitude in the control and in the presence of ryanodine. Ryanodine caused no significant change in SEJP frequency, amplitude, or amplitude distribution. In all traces, RMPs were between −70 and −80 mV.

It has previously been shown that nicotine-induced Ca^2+^ transients in nerve terminals in the mouse vas deferens depend on Ca^2+^-induced Ca^2+^ release (CICR) from intraneuronal Ca^2+^ stores [14]. To establish the role of CICR in the generation of epibatidine-induced neurotransmitter release, the effect of ryanodine on EID generation was investigated. In the presence of ryanodine, EIDs were significantly altered (Fig. 4A). While the proportion of responding cells was not significantly different from controls (proportion control = 82±7%, proportion cells ryanodine = 70±7%; *P* = 0.14; *n_c_* control = 44, *n_c_* ryanodine = 46 *n_v_* = 5), there was a significant reduction in the frequency of EIDs in the presence on ryanodine: from 1.44±0.08 Hz in the control to 0.92±0.09 Hz in the presence of ryanodine (*P*<0.05; *n_c_* control = 30, *n_c_* ryanodine = 30 *n_v_* = 5). Surprisingly, the mean amplitude of EIDs significantly decreased from 10.1±0.5 mV in the control, to 6.0±0.5 mV in the presence of ryanodine (*P*<0.05). There was also a large shift in EID amplitude distribution (*P*<0.05; Fig. 4B).

The reduction in amplitude of EIDs in the presence of ryanodine is consistent with either a prejunctional or a postjunctional effect. To investigate the potential postjunctional effect of ryanodine and/or the contribution of CICR to low frequency EJPs, the effect of ryanodine on EJP amplitude was investigated. As with the previous ryanodine experiments, preparations were exposed to ryanodine while stimulated (10 stimuli at 10 Hz every 30 s for 60 min). There was no detectable effect on mean EJP amplitude: following treatment with 10 µM ryanodine, the amplitude of the first EJP in a pair, 200 ms apart, evoked at frequency 0.2 Hz was 88.5±7% of controls (*P* = 0.10, unpaired Student's *t*-test; *n_c_* control = 36, *n_c_* ryanodine = 36, *n_v_* = 6; Fig. 4C). Previous studies have suggested that, in some preparations, ryanodine-sensitive Ca^2+^ stores may be involved in facilitation of neurotransmitter release [18]. Ryanodine had no effect on paired-pulse facilitation of EJP amplitude. In the presence of ryanodine, paired-pulse facilitation of EJPs was 98±2% of the control facilitation (*P* = 0.41, unpaired Student's *t*-test; Fig. 4C).

A postjunctional effect of ryanodine has the potential to change the time course of decay of the EJP, for example by a direct effect on the P2X receptor. There was no significant effect of ryanodine on the time course of repolarisation (decay time constant): the average time taken for the membrane potential to fall from 90% to 33% (i.e. by about 1/e) of the peak amplitude of an EJP was 39±1 ms in the control; in the presence of ryanodine the decay time was 36±2 ms (*P* = 0.10, unpaired Student's *t*-test).

Ryanodine had no effect on the mean frequency of occurrence of SEJPs (Fig. 4D). In the presence of ryanodine (60 min), the frequency of SEJP occurrence was 94±13% of the control (*P* = 0.85). A postjunctional effect of ryanodine would be detected by a change in the amplitude of SEJPs, but in these experiments no significant change in amplitude was observed: control amplitude = 4.9±0.3 mV, ryanodine amplitude = 4.5±0.3 (*P* = 0.29, unpaired Student's *t*-test). There was no significant difference between SEJP amplitude distributions recorded in the presence and absence of ryanodine (*P* = 0.12; Fig. 4E). These results indicate that EIDs are partially dependent on CICR, whereas electrically-evoked neurotransmitter release (at low frequencies of stimulation) and spontaneous neurotransmitters release do not involve CICR.

## Discussion

This study has shown that rapid application of epibatidine caused a series of smooth muscle cell depolarisations similar in appearance to SEJPS that were sensitive to nonselective nAChR antagonists and blocked following desensitisation of P2X1 receptors. The most plausible explanation for these depolarisations is that activation of prejunctional nAChRs triggers neurotransmitter (ATP) release from sympathetic nerve terminals. We have previously shown that ‘rapid’ (but not local) bath application of epibatidine causes an increase in neurotransmitter release from nerve terminals in mouse vasa deferentia [15]. The rapid, local application method described in the present study enables multiple recordings of epibatidine-induced response to be made from the same preparation. This method greatly facilitates the investigation of the mechanism of epibatidine-induced neurotransmitter release in the mouse vas deferens.

### Epibatidine-induced neurotransmitter release occurred following activation of non-α7 nAChRs

EIDs could be elicited in the presence of α-BTX or MLA at concentrations which have previously been demonstrated to block α7-nAChR-mediated neurotransmitter release [7], [19], [20]. These findings suggest that the majority of epibatidine-induced neurotransmitter release follows activation of nAChR receptors which do not contain α7 subunits. Additionally, epibatidine has a relatively low potency at α7 nAChRs in comparison to other neuronal nAChRs [21] and given that the actual concentration of epibatidine that reached the nerve terminals is likely to be substantially less than 1 µM, it is possible that the concentration of epibatidine that reaches the nerves terminals will be insufficient to activate α7-containing nAChRs that may be present.

In the presence of α-BTX and MLA, there was a small shift of EIDs to lower amplitudes, suggesting that there may be a small proportion of epibatidine-induced neurotransmitter release that does depend on α7-nAChR activation. Previous investigations of nAChR agonist-induced neurotransmitter release have shown partial sensitivity to α-BTX; for example, in rat vas deferens, a mixture of α3β4 and α7 nAChRs are expressed [22]. The majority of epibatidine-induced neurotransmitter release appeared to be independent of α7-nAChR. A number of other examples of nAChR agonist-induced neurotransmitter release that are α-BTX insensitive have been described in other sympathetic neurons [23].

The particular subtype of nAChRs responsible for EID generation remains to be determined. Of the non-α7 nAChRs present in mouse sympathetic postganglionic neurons, the vast majority are α3β4, α3β4α5 or α3β4β2 [24], so it is likely that one of these subtypes is involved. Other studies have described a role for α5 nAChRs in neurotransmitter release from axonal sprouts of mouse cultured superior cervical neurons [25], but speculation on the subtype based on these results is difficult because of different nAChR subtype distribution even within the same sympathetic neuron [23], [25].

### Generation of nerve terminal action potentials is not required for epibatidine-induced neurotransmitter release

nAChR-induced TTX-resistant release of neurotransmitter is well established in cultured sympathetic neurons [23], [26], [27]. nAChRs are ligand-gated cation channels [28], so an influx of cations through nAChRs might initiate neuronal action potentials leading to neurotransmitter release. In the present study, however, EIDs could still be elicited in the presence of TTX, although there was a small reduction in their mean amplitude (83±4% of the control). These results suggest that a small proportion of epibatidine-induced neurotransmitter release depends on action potential initiation, but most of the neurotransmitter release does not require functional voltage-gated Na^+^ channels (VGSCs). Previous results from this laboratory have shown a TTX-insensitive component of epibatidine-induced neurotransmitter release in contraction studies [15] and demonstrated that nicotine-induced Ca^2+^ transients in nerve terminals in the mouse vas deferens were unaffected by TTX [14].

### The role of VGCCs in epibatidine-induced neurotransmitter release

The experiments carried out in the presence of Cd^2+^ indicate that most of the epibatidine-induced neurotransmitter release is independent of VGCC activation. In the present work, the proportion of Cd^2+^-sensitive neurotransmitter release is similar to the proportion of TTX-sensitive neurotransmitter release (Fig. 3). Assuming that TTX-sensitive neurotransmitter release requires VGCC activation, it is likely that the Cd^2+^- and TTX-sensitive release represent the same proportion of epibatidine-induced neurotransmitter release.

### Epibatidine-induced neurotransmitter release is dependent on extracellular Ca^2+^

When [Ca^2+^]_o_ was reduced from 1.8 mM to 0.6 mM, there was a significant reduction in the frequency of occurrence of EIDs. A small decrease in EID amplitude may be expected in the presence of reduced [Ca^2+^]_o_, because about 12% of the current through heterologously expressed P2X1 receptors is carried by Ca^2+^ [29], but the present study was not sufficiently powered to detect this effect. It has been demonstrated that influx of Ca^2+^ through nAChRs is sufficient to cause an increase in the intraterminal Ca^2+^ concentration in this preparation [14].

### Intraneuronal Ca^2+^ stores and epibatidine-induced neurotransmitter release

The largest decrease of mean EID amplitude and change of amplitude distribution was caused by ryanodine. Ryanodine also decreased the frequency of occurrence to 64±4% of the control. The ryanodine treatment method used here depletes intraneuronal stores and prevents CICR [30]. Activation of nAChRs can cause mobilisation of intraneuronal Ca^2+^ stores in sympathetic nerves innervating the mouse vas deferens [14], somatic spines emanating from chick ciliary ganglion neurones [31] and in SH-SY5Y cells [32]. Ryanodine-sensitive Ca^2+^ stores have also been shown to have a role in nAChR-induced neurotransmitter release: ryanodine reduces action potential-independent nicotine-induced glutamate release [8] and nicotine-induced potentiation of electrically-evoked glutamate release in rat hippocampus [33]. Previous studies demonstrating the involvement of intraneuronal Ca^2+^ stores in nAChR-induced neurotransmitter release have implicated the α7 nAChRs, (e.g. [34], [35]; see references above). Interestingly, in our experiments, α7 nAChR did not appear to have a substantial role in epibatidine-induced neurotransmitter release. These findings indicate that an alternative, as yet unidentified, subtype of nAChR can couple to CICR and neurotransmitter release. Further investigation of the role of these intraterminal Ca^2+^ stores with SERCA inhibitors would be of great interest.

### nAChR activaction may cause multiquantal neurotransmitter release following mobilisation of intraterminal Ca^2+^ stores

The reduction of the mean amplitude of EIDs may have been caused by an inhibition of multipacketed release, or near-synchronous packeted release, from the same varicosity. CICR-dependent, multiquantal neurotransmitter release following nAChR activation has been demonstrated in the CA3 region of the rat hippocampus. Nicotine elicited high amplitude multiquantal spontaneous EPSCs which were abolished by ryanodine and thapsigargin [8]. In the mouse isolated vas deferens, nicotine and epibatidine induce ryanodine-sensitive Ca^2+^ transients in nerve terminals [14]. It is possible that these nAChR-induced Ca^2+^ transients, similar to the spontaneous Ca^2+^ transients described by Llano *et al.* [36], could result in multivesicular release and be responsible for the generation of high-amplitude EIDs. An alternative mechanism is that high-amplitude EIDs may result from summation of ATP release from several varicosities. Since ryanodine reduced the overall frequency of occurrence of EIDs, the probability of stochastic simultaneous occurrences and therefore the number of high-amplitude EIDs would decrease. This mechanism appears less likely because at low [Ca^2+^]_o_ there is a greater reduction in EID frequency than with ryanodine treatment, but low [Ca^2+^]_o_ did not significantly affect the mean EID amplitude.

While there was a clear effect of ryanodine on epibatidine-induced neurotransmitter release, ryanodine had no significant effect on electrically-evoked or spontaneous neurotransmitter release. It is possible that CICR, caused by an influx of Ca^2+^ following an action potential, whilst being too slow to influence neurotransmitter release following that action potential, could facilitate neurotransmitter release evoked by the next action potential in a train. In the CNS, the effect of CICR on paired-pulse facilitation is unclear (see [37]; for a review). In the present study, ryanodine did not change the degree of potentiation of pairs of EJPs evoked at 5 Hz. These results are supported by previous studies in the rodent vas deferens: ryanodine-sensitive neurotransmitter release is only revealed following trains of stimuli at high frequency (3–50 Hz) in the presence of ω-conotoxin GVIA to block N-type VGCCs [30]; in nerve terminals in the mouse vas deferens, low frequency action potential-evoked Ca^2+^ transients were not affected by ryanodine [38]. In the present investigation, ryanodine had no effect on spontaneous neurotransmitter release. Previous studies in the CNS have shown that most of the spontaneous neurotransmitter release that is sensitive to ryanodine is multivesicular or occurs as bursts of neurotransmitter release [37]. It is possible that this type of spontaneous neurotransmitter release occurs very infrequently in the mouse vas deferens and could not be detected in present experiments.

### The tissue location of nAChRs

While a number of different cell types are present in the mouse vas deferens, including a small number of cholinergic nerve terminals which also contain prejunctional nAChRs [39], the insensitivity of EIDs to TTX/Cd^2+^, the highly local agonist application and the rapid onset of neurotransmitter release, all suggest that the nAChRs responsible for the observed neurotransmitter release are located directly on the sympathetic nerve terminals. Despite this, we are unable to rule out rapid signaling to sympathetic terminals from nicotinic receptors located on nearby cells.

Of the cells monitored, 24% did not respond to epibatidine. Care was taken to record from cells positioned close to the surface of the vas deferens, adjacent to the pipette. Unfortunately, it was not possible to determine accurately the position of the impaled cell and some recordings were likely to have been obtained from cells located deeper within the tissue. Given the small volume of epibatidine applied and the transient nature of its application, it is possible the lack of response was caused by an insufficient concentration of epibatidine reaching nerve terminals that innervated these cells. Bath application of 100 nM epibatidine causes robust and reliable neurotransmitter release [15], which indicates that in the ‘non-responders’ in current study, the concentration of epibatidine at the nerve terminals was likely to be substantially less than 100 nM. Variability in the epibatidine concentration that reaches the nerve terminals may also be responsible for the variability in the size of the epibatidine-induced response. The alternative hypothesis, that terminals possess differing numbers of nAChRs, cannot be ruled out.

### Physiological and pathophysiological relevance of nAChRs on sympathetic nerve terminals

Given that the vast majority of sympathetic postganglionic neurons do not use acetylcholine as a transmitter, the physiological function of prejunctional nicotinic receptors remains unclear and may only rarely be functionally important. While it is true that sympathetic and parasympathetic axons and terminals can run in close proximity within the peripheral organs (for an example in the mouse vas deferens, see [40]), the common presence of inhibitory muscarinic receptors on sympathetic nerve terminals [41], which have a much higher affinity for acetylcholine, implies that only exposure to high local concentrations of acetylcholine are likely to produce a nicotinic excitatory response that is sufficient to oppose the muscarinic inhibition. It is also possible that the sympathetic nerves are sensitive to local non-neuronal acetylcholine sources [42]. Given that only provisional reports link endogenous acetylcholine release (acting at prejunctional nicotinic receptors) to an increased release of noradrenaline from sympathetic terminals [43], at present the most important implications for the current study are in the peripheral response to drugs and as a model system for understanding nerve terminal Ca^2+^ regulation in the context of nicotinic receptor activation.

Nicotine's actions on sympathetic nerve terminals contribute to its acute cardiovascular effects [4], [44], [45], and may also play a role in sympathetic innervation of the urogenital tract. For example, acute nicotine exposure (nicotine gum) reduces erectile function without affecting subjective sexual arousal [46], which points towards a peripheral amplification of sympathetic drive, noting that sympathetic drive to the penis is associated with flaccidity.

Central nervous system presynaptic nicotinic receptors remain key targets for pharmacological approaches to dementias, although the currently available drugs have produced only marginal clinical improvements [47]; hence, understanding tractable models of nicotinic receptor function is useful for drug development.

In summary, this study describes a nAChR-induced neurotransmitter release of which the majority is independent of neuronal action potential generation and does not require VGCCs. The most likely mechanism is a combination of influx of Ca^2+^ through nAChRs and CICR from ryanodine-sensitive intraneuronal Ca^2+^ stores. The unidentified nAChR subtype responsible for this neurotransmitter release is one of the first descriptions of a non-α7 nAChR that can couple to CICR and subsequent neurotransmitter release.

## Supporting Information

Figure S1 **Time courses of SEJP and EID.** Representative membrane trace of an EID (black line) and a SEJP (thick grey line) obtained from the same recording 10 s before epibatidine application (SEJP) and 3 s after epibatidine application (EID). The amplitude of the SEJP and EID were normalized to facilitate comparison of the time courses.
(TIF)

Figure S2 **Variable responses to epibatidine application.** Membrane potential traces from three different cells (A–C) before and after 1 µM epibatidine application. The time of epibatidine application is indicated by an arrow. Resting membrane potentials were −70 to −80 mV.
(TIF)

Figure S3 **Time course of frequency of EID occurrence in the presence of various drugs.** Each point represents the mean frequency of SEJP/EID occurrence collected in 2 s bins. The error bars represent the s.e.m. Only data from responding cells are included. Control recordings are shown in blue and recordings in the presence of a drug are shown in red. A) Time control (*n_c_* control = 29, *n_c_* time control = 30, *n_v_* = 6). B) 100 nM α-bungarotoxin (α-BTX; *n_c_* control = 30, *n_c_* α-BTX = 31, *n_v_* = 5). C) 20 nM methyllacaconitine (MLA; *n_c_* control = 30, *n_c_* MLA = 30, *n_v_* = 5). D) 300 nM tetrodotoxin (TTX;, *n_c_* control = 29, *n_c_* TTX = 31, *n_v_* = 6). E) 100 µM Cd^2+^ (*n_c_* control = 28, *n_c_* Cd^2+^ = 30, *n_v_* = 5). F) 0.6 mM extracellular Ca^2+^ (0.6 mM [Ca^2+^]_o_; *n_c_* control = 30, *n_c_* 0.6 mM [Ca^2+^]_o_, *n_v_* = 5). G) 10 µM ryanodine (*n_c_* control = 30, *n_c_* ryanodine = 30, *n_v_* = 5).
(TIF)
