# Supplementary figures and images for: Mechanisms Involved in Nicotinic Acetylcholine Receptor-Induced Neurotransmitter Release from Sympathetic Nerve Terminals in the Mouse Vas Deferens
Source: PLoS One. 2011 Dec 22;6(12):e29209. doi: 10.1371/journal.pone.0029209 (PMC3245264; doi:10.1371/journal.pone.0029209)

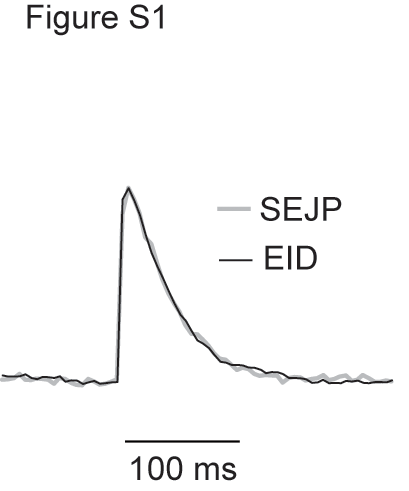

Supplement: Figure S1 — Time courses of SEJP and EID. Representative membrane trace of an EID (black line) and a SEJP (thick grey line) obtained from the same recording 10 s before epibatidine application (SEJP) and 3 s after epibatidine application (EID). The amplitude of the SEJP and EID were normalized to facilitate comparison of the time courses. (TIF) [file pone.0029209.s001.tif]

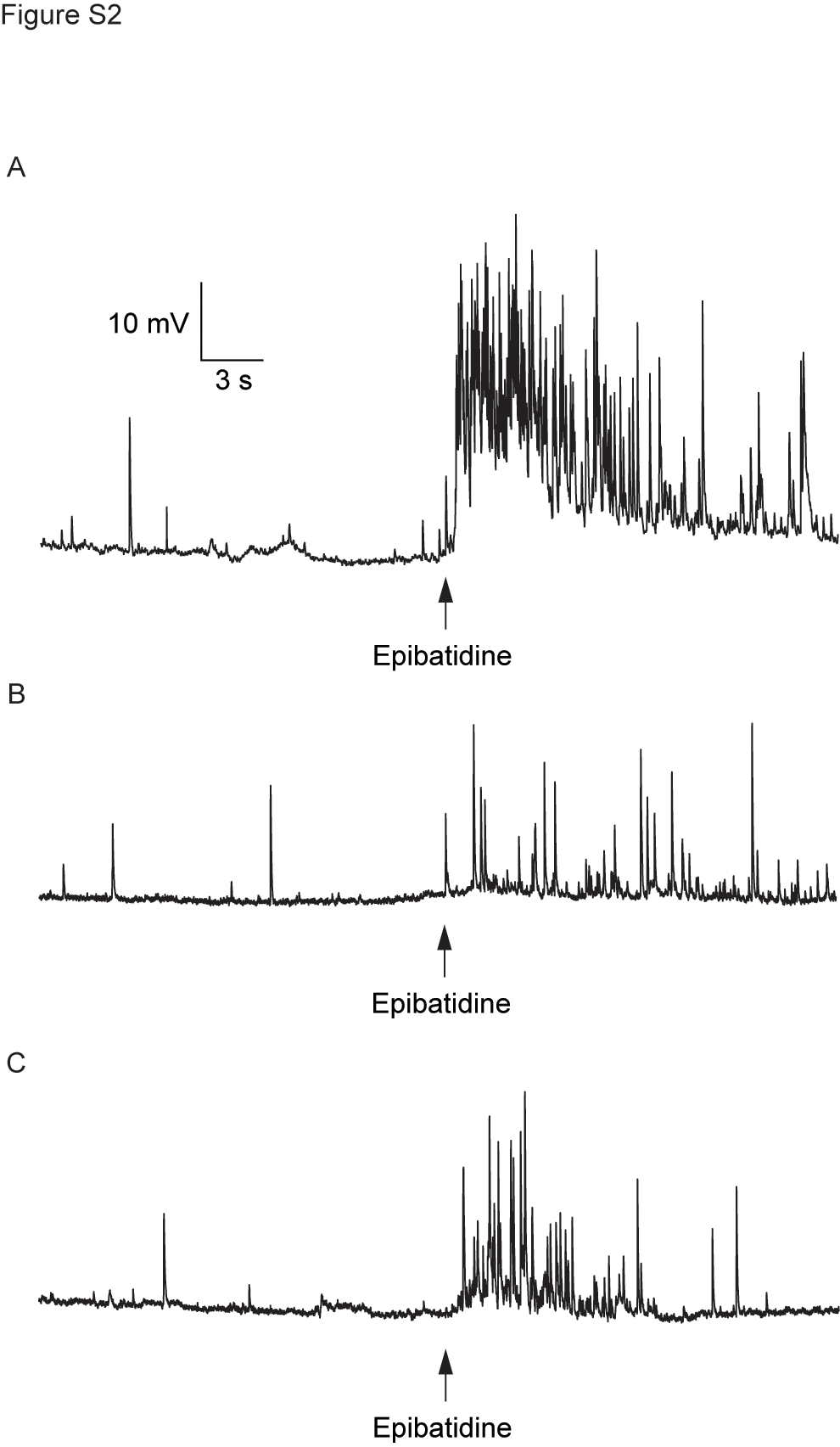

Supplement: Figure S2 — Variable responses to epibatidine application. Membrane potential traces from three different cells (A–C) before and after 1 µM epibatidine application. The time of epibatidine application is indicated by an arrow. Resting membrane potentials were −70 to −80 mV. (TIF) [file pone.0029209.s002.tif]

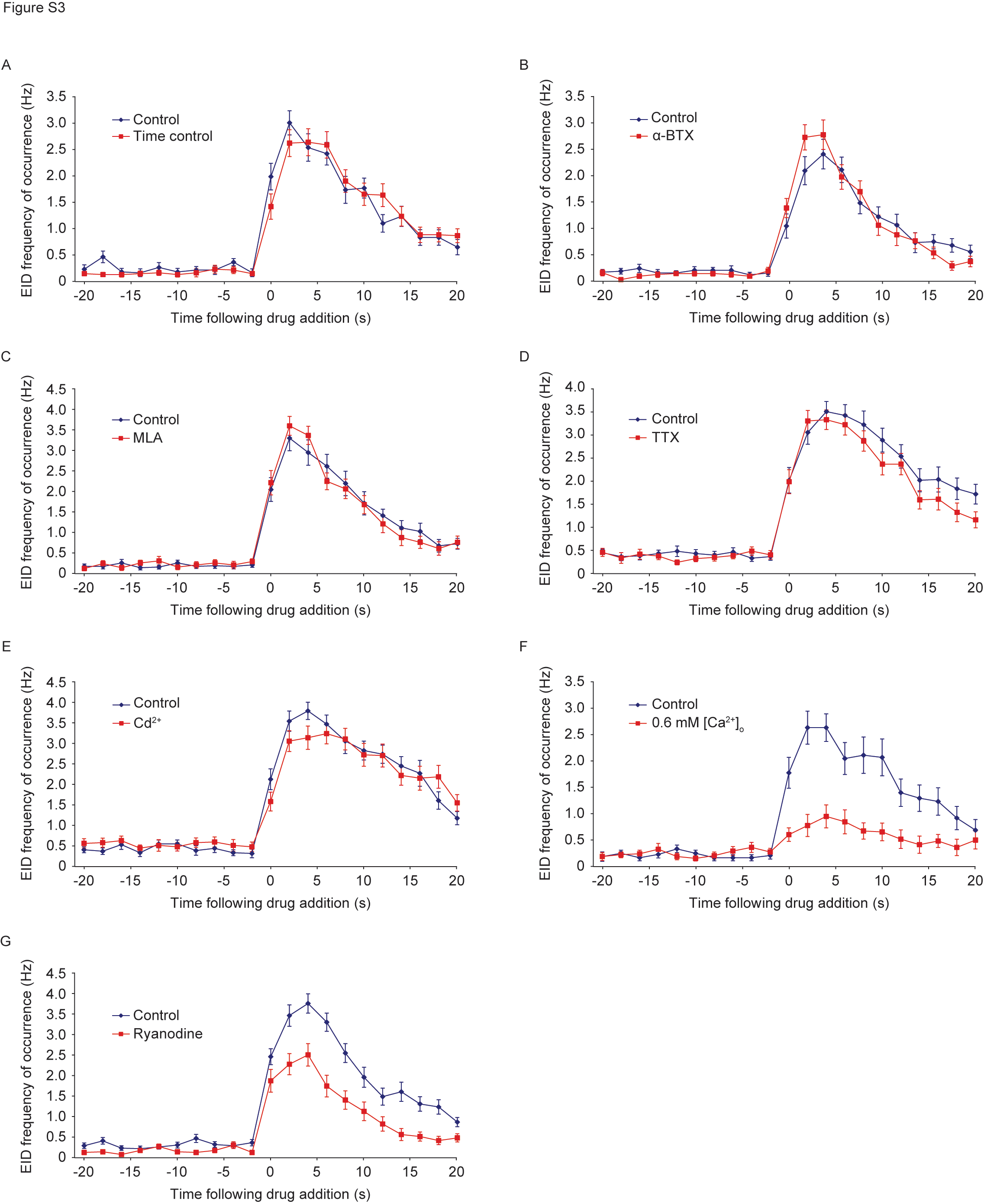

Supplement: Figure S3 — Time course of frequency of EID occurrence in the presence of various drugs. Each point represents the mean frequency of SEJP/EID occurrence collected in 2 s bins. The error bars represent the s.e.m. Only data from responding cells are included. Control recordings are shown in blue and recordings in the presence of a drug are shown in red. A) Time control (nc control = 29, nc time control = 30, nv = 6). B) 100 nM α-bungarotoxin (α-BTX; nc control = 30, nc α-BTX = 31, nv = 5). C) 20 nM methyllacaconitine (MLA; nc control = 30, nc MLA = 30, nv = 5). D) 300 nM tetrodotoxin (TTX;, nc control = 29, nc TTX = 31, nv = 6). E) 100 µM Cd2+ (nc control = 28, nc Cd2+ = 30, nv = 5). F) 0.6 mM extracellular Ca2+ (0.6 mM [Ca2+]o; nc control = 30, nc 0.6 mM [Ca2+]o, nv = 5). G) 10 µM ryanodine (nc control = 30, nc ryanodine = 30, nv = 5). (TIF) [file pone.0029209.s003.tif]
